# Raw Cow’s Milk Prevents the Development of Airway Inflammation in a Murine House Dust Mite-Induced Asthma Model

**DOI:** 10.3389/fimmu.2017.01045

**Published:** 2017-08-28

**Authors:** Suzanne Abbring, Kim A. T. Verheijden, Mara A. P. Diks, Athea Leusink-Muis, Gert Hols, Ton Baars, Johan Garssen, Betty C. A. M. van Esch

**Affiliations:** ^1^Division of Pharmacology, Faculty of Science, Utrecht Institute for Pharmaceutical Sciences, Utrecht University, Utrecht, Netherlands; ^2^Nutricia Research, Utrecht, Netherlands; ^3^Research Institute of Organic Agriculture (FiBL), Frick, Switzerland

**Keywords:** allergy, asthma, farming effect, house dust mite, milk processing, mouse models, raw milk

## Abstract

Epidemiological studies show an inverse relation between raw cow’s milk consumption and the development of asthma. This protective effect seems to be abolished by milk processing. However, evidence for a causal relationship is lacking, and direct comparisons between raw and processed milk are hardly studied. Therefore, this study investigated the preventive capacity of raw and heated raw milk on the development of house dust mite (HDM)-induced allergic asthma in mice. Six- to seven-week-old male BALB/c mice were intranasally (i.n.) sensitized with 1 µg HDM or PBS on day 0, followed by an i.n. challenge with 10 µg HDM or PBS on days 7–11. In addition, mice were fed 0.5 mL raw cow’s milk, heated raw cow’s milk, or PBS three times a week throughout the study, starting 1 day before sensitization. On day 14, airway hyperresponsiveness (AHR) in response to increasing doses of methacholine was measured to assess lung function. Bronchoalveolar lavage fluid (BALF) and lungs were furthermore collected to study the extent of airway inflammation. Raw milk prevented both HDM-induced AHR and pulmonary eosinophilic inflammation, whereas heated raw milk did not. Both milk types suppressed the Th2-polarizing chemokine CCL17 in lung homogenates and reduced lung Th2 and Th17 cell frequency. IL-4 and IL-13 production after *ex vivo* restimulation of lung T cells with HDM was also reduced by both milk types. However, local IL-5 and IL-13 concentrations were only suppressed by raw milk. These findings support the asthma-protective capacity of raw cow’s milk and show the importance of reduced local type 2 cytokine levels. Heated raw milk did not show an asthma-protective effect, which indicates the involvement of heat-sensitive components. Besides causal evidence, this study provides the basis for further mechanistic studies.

## Introduction

With up to 300 million people affected worldwide, asthma is a global burden (1). It is one of the most common chronic diseases in the world, and the prevalence has increased significantly in Western countries in the last decades (2). The disease is characterized by airway hyperresponsiveness (AHR), eosinophilic airway inflammation, and excessive mucus secretion. This leads to shortness of breath, wheezing, coughing, and chest tightness in genetically susceptible individuals. Asthma can be triggered by a wide range of environmental stimuli, such as inhaled allergens [e.g., house dust mite (HDM), animal dander], respiratory infections, and airborne pollutants (e.g., tobacco smoke, air pollution) (3).

The remarkable increase in the prevalence of asthma and allergies in Western countries in recent decades is often explained by the loss of rural living conditions and associated changes in diet and lifestyle (4). This so-called hygiene hypothesis is supported by numerous epidemiological studies that consistently show that growing up on a farm reduces the risk of developing asthma and allergies (5–8). Farm-associated exposures contributing to this protective effect are contact with livestock, contact with animal feed, and the consumption of raw, unprocessed, cow’s milk (9–13). The protective effect of raw cow’s milk consumption is of particular interest since this was found to be independent of concomitant farm exposures and farm status (10, 12, 13). The latter suggests that non-farming populations might also benefit from the protective effects of raw cow’s milk consumption.

Interestingly, cross-sectional evidence suggests that the asthma and allergy protective effects of raw cow’s milk are abolished by milk processing (9). Processed cow’s milk used for commercial purposes differs in many respects from raw cow’s milk. Commercial milk is usually homogenized and heat treated to increase shelf life and to inactivate potentially pathogenic microorganisms (14). However, this milk processing also affects other milk components. The structure of the milk fat, for example, is changed remarkably by homogenization, and this might alter allergen presentation (15). In addition, heat-sensitive milk components such as proteins can be structurally altered upon heating and consequently lose functionality (16). These changes might (partly) explain why the beneficial farm milk effect seems to be lost after processing (9).

At the moment, raw milk can theoretically be considered as preventive treatment, but in reality, its consumption is limited due to its possible contamination with pathogens. To produce safe and protective milk, components and mechanisms underlying the asthma-protective effect need to be elucidated. At the same time, the current epidemiological evidence needs to be confirmed by causality. In the present study, we therefore examined the ability of raw cow’s milk consumption to prevent the development of asthma in a murine HDM-induced asthma model. Because many differences between raw milk and commercial milk make it difficult to disentangle the different effects of milk processing, we compared raw and heated raw cow’s milk to solely assess the effects of heating.

## Materials and Methods

### Mice

Six- to seven-week-old, specific pathogen-free, male BALB/c mice (*n* = 9/group) were purchased from Charles River Laboratories (The Netherlands) and housed at the animal facility of the Utrecht University under bio-contained sterile conditions using HEPA^®^ filtered isocages^®^ (Techniplast, Italy). Food and water were provided *ad libitum*. All animal procedures were conducted in accordance with the governmental guidelines and approved by the Ethical Committee for Animal Research of the Utrecht University, Utrecht, The Netherlands (DEC2014.III.12.117).

### HDM-Induced Asthma Model

**Figure 1:**
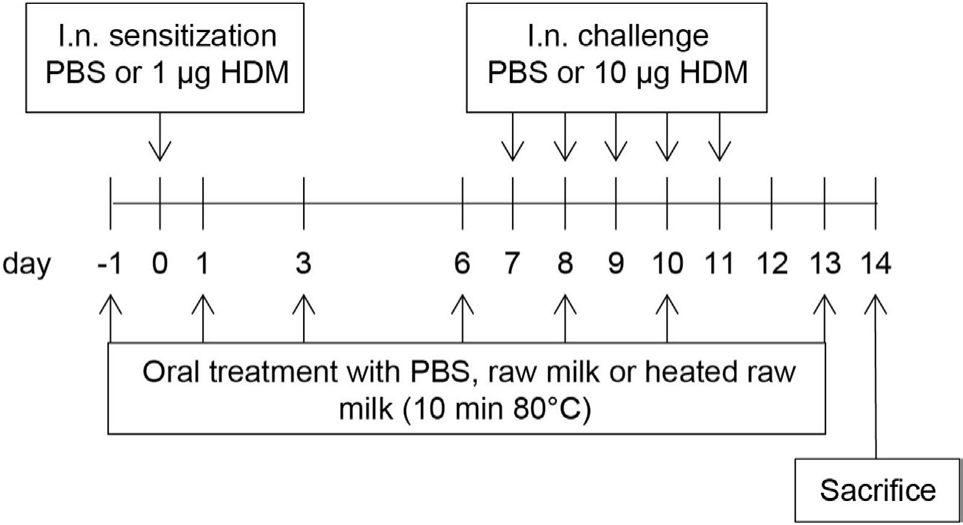
Schematic overview of the study design. Male BALB/c mice (*n* = 9/group) were sensitized intranasally (i.n.) with PBS or house dust mite (HDM) on day 0 and were challenged i.n. on days 7–11 with PBS or HDM. By oral gavage, the mice were given raw cow’s milk, heated raw cow’s milk, or PBS three times a week throughout the study, starting 1 day before sensitization. Mice were killed on day 14.

A schematic overview of the experimental design is shown in Figure 1. Anesthetized mice were intranasally (i.n.) sensitized with PBS or 1 µg HDM/40 μl PBS (Greer Laboratories, USA) on day 0 followed by an i.n. challenge with PBS or 10 µg HDM/40 μl PBS on days 7–11 (17). Three times a week throughout the study, starting 1 day before sensitization, mice were fed 0.5 mL raw cow’s milk, heated raw cow’s milk, or PBS by oral gavage. Raw cow’s milk was collected from a biodynamic dairy farm (Hof Dannwisch, Germany). One day after collection, part of the raw milk was aliquoted and stored at −20°C until further use. The remainder was heated for 10 min at 80°C in a water bath, allowed to cool to room temperature, aliquoted, and then stored at −20°C until further use. Mice were killed on day 14.

### Airway Responsiveness

Mice were intraperitoneally (i.p.) anesthetized with a mixture of ketamine (Vetoquinol S.A., France; 125 mg/kg) and medetomidine (Pfizer, The Netherlands; 0.4 mg/kg). EMKA invasive measurement of dynamic resistance (EMKA Technologies, France) in response to increasing doses of methacholine (acetyl-β-methyl-choline chloride, Sigma-Aldrich, The Netherlands; 0–25 mg/mL, 10% puff for 10 s.) was used on day 14 to assess lung function. Average lung resistance (R_L_) is presented in cm H_2_O/(mL/sec) (18).

### Bronchoalveolar Lavage

On day 14, mice were killed using an i.p. overdose of pentobarbital (Nembutal™, Ceva Santé Animale, The Netherlands; 600 mg/kg). Lungs were lavaged through a tracheal cannula with 1 mL of pyrogen-free saline (0.9% NaCl, 37°C) supplemented with protease inhibitor cocktail tablet (Complete Mini, Roche Diagnostics, Germany). This was followed by three lavages with 1 mL saline solution (0.9% NaCl, 37°C). The bronchoalveolar lavage fluid (BALF) was centrifuged (400 g, 5 min), and pellets of the four lavages were pooled. Total numbers of BALF cells were counted using a Bürker-Türk chamber (magnification 100×). For differential BALF cell counts, cytospin preparations were made and stained with Diff-Quick (Merz & Dade A.G., Switzerland). Numbers of eosinophils, neutrophils, lymphocytes, and macrophages were scored using light microscopy. At least 200 cells were counted, and the absolute number of each cell type was calculated (19).

### Preparation of Lung Homogenates

Lung samples were homogenized in 1% Trition X-100 (Sigma-Aldrich)/PBS containing protease inhibitor (Complete Mini, Roche Diagnostics) using a Precellys 24 tissue homogenizer (Bertin Technologies, France). Homogenates were centrifuged (14.000 rpm, 10 min), and supernatants were collected and stored at −20°C until further use. Prior to cytokine and chemokine analysis, the protein concentration of each sample was determined by using the Pierce BCA protein assay kit standardized to bovine serum albumin (BSA) according to the manufacturer’s protocol (Thermo Fisher Scientific, USA). Homogenates were subsequently diluted to a final concentration of 1 mg protein/mL.

### *Ex Vivo* Lung Restimulation with HDM

Single-lung cell suspensions were obtained by cutting lung samples into small pieces and by adding digestion buffer containing DNase I and Collagenase A (Roche Diagnostics) for 30 min. The digestion was stopped by adding fetal bovine serum (FBS; Bodinco, The Netherlands). Lung pieces were passed through a 70-µm nylon cell strainer and rinsed with 10 mL RPMI. Cells were washed and resuspended in RPMI 1640 culture medium (Lonza, USA) supplemented with 10% heat-inactivated FBS and 0.1% penicillin–streptomycin solution (Sigma-Aldrich). Lung cells (4 × 10^5^ cells/well) were cultured in medium with or without 50 µg/mL HDM. Supernatant was harvested after 4 days of culture (37°C, 5% CO_2_) and stored at −20°C until further analysis.

### Measurement of Cytokines and Chemokines

IL-33, CCL20, CCL17, and CCL22 concentrations were measured in lung homogenates with a DuoSet ELISA (R&D Systems, USA) and IL-5 and IL-13 with a Ready-SET-Go!^®^ ELISA (eBioscience, USA). A standard IL-13 flex set or a standard Th1/Th2/Th17 assay (BD Biosciences, The Netherlands) was used to determine cytokine concentrations in supernatants of lung restimulation. All assays were performed according to the manufacturer’s protocol. The concentrations of cytokines and chemokines were expressed as picogram per milligram protein in lung homogenates and picogram per milliliter in lung restimulation supernatants.

### Flow Cytometric Analysis of Immune Cells in the Lung

Single-lung cell suspensions were resuspended in PBS/1% BSA (Sigma-Aldrich) and incubated for 15 min at 4°C with Fc block CD16/CD32 antibodies (BD Biosciences; 5 µg/mL) to prevent non-antigen-specific binding. Cells were subsequently stained with antibodies (eBioscience, The Netherlands, unless otherwise stated) against CD4-PerCP Cy5.5, GATA3-PE, Tbet-eFluor^®^ 660, RORγt-PE, CD25-Alexa Fluor^®^ 488, FoxP3-APC, CD11c-PerCP Cy5.5, MHCII-PE Cy7, CD11b-PE, and Fixable Viability Dye-eFluor^®^ 780 or matching isotype controls for 30 min at 4°C. Cells were fixed using fixation buffer (eBioscience) or permeabilized for intracellular staining using the FoxP3 staining buffer set (eBioscience) according to the manufacturer’s protocol. Flow cytometry was performed using FACS Canto II (BD Biosciences), and results were analyzed using Flowlogic Software (Inivai Technologies, Australia). To distinguish between negative and positive staining cell populations, fluorescence minus one controls were used (after the exclusion of debris, doublets and non-viable cells).

### Statistical Analysis

Data are presented as mean ± SEM and were analyzed using one- or two-way ANOVA, followed by Bonferroni’s multiple comparisons test. As pulmonary IL-5 concentrations did not obtain normality, data were analyzed using Kruskal–Wallis test followed by Dunn’s multiple comparisons test. Results were considered statistically significant when *P* < 0.05. Analyses were performed using GraphPad Prism software (version 6.07).

## Results

### Raw Milk Prevents HDM-Induced AHR and Pulmonary Inflammation

**Figure 2:**
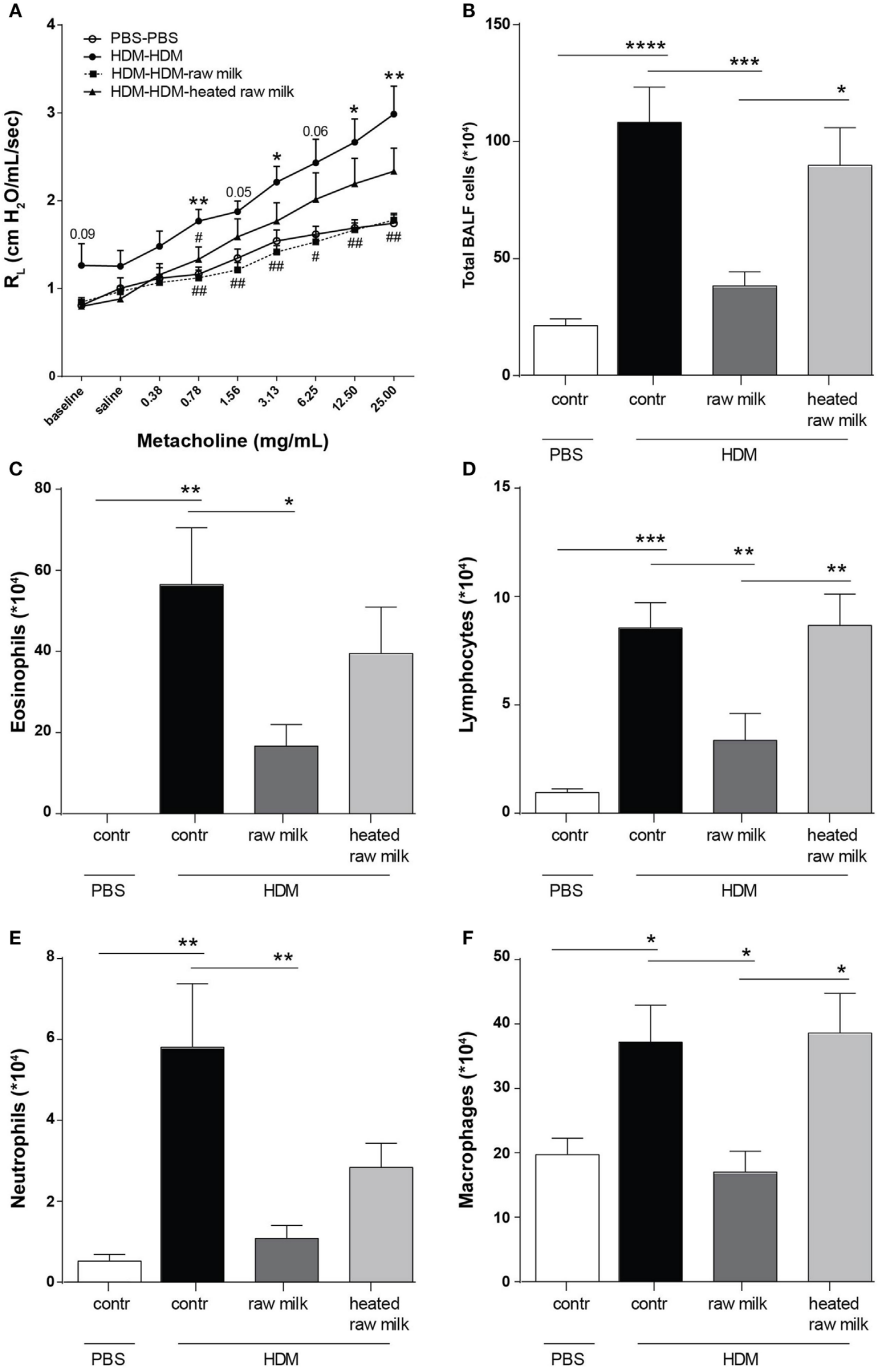
House dust mite (HDM)-induced airway hyperresponsiveness and pulmonary inflammation were prevented by raw milk. **(A)** Lung resistance (R_L_) measured after exposure to increasing doses of methacholine. **(B–E)** Differential bronchoalveolar lavage fluid (BALF) cell counts. **(B)** Total BALF cells, **(C)** absolute number of eosinophils, **(D)** lymphocytes, **(E)** neutrophils, and **(F)** macrophages. Data are presented as mean ± SEM. (**A**) *n* = 7–9 mice/group. *P < 0.05, **P < 0.01 compared to PBS-PBS group, ^#^*P* < 0.05, ^##^*P* < 0.01 compared to HDM-HDM group as analyzed with one-way ANOVA followed by Bonferroni’s multiple comparisons test. (**B–E**) *n* = 8–9 mice/group. *P < 0.05, **P < 0.01, ***P < 0.001, ****P < 0.0001 as analyzed with one-way ANOVA followed by Bonferroni’s multiple comparisons test. Contr, mice treated with PBS.

To assess the effects of raw and heated raw milk on lung function, AHR was measured in response to increasing doses of methacholine. Baseline airway responsiveness tended to be higher in HDM-mice compared to PBS-mice. An increase in AHR was observed in HDM-mice compared to PBS-mice after methacholine exposure. Although intervention with heated raw milk showed no significant effect, increased AHR was prevented by intervention with raw milk (Figure 2A). To study the extent of pulmonary inflammation, BALF was examined. The total number of inflammatory cells in the BALF of HDM-mice was significantly higher than in PBS-mice (Figure 2B). This was mainly due to an increase in the number of eosinophils (Figure 2C), although numbers of lymphocytes, neutrophils, and macrophages were increased as well (Figures 2D–F). Raw milk reduced the total number of inflammatory cells in the BALF, which was reflected by lower numbers of eosinophils, lymphocytes, neutrophils, and macrophages (Figures 2B–F). Pulmonary inflammation was unaffected by heated raw milk (Figures 2B–F).

### Raw Milk Suppresses the Frequency of Conventional Dendritic Cells (cDCs) and Related CCL17 Concentrations in the Lung

**Figure 3:**
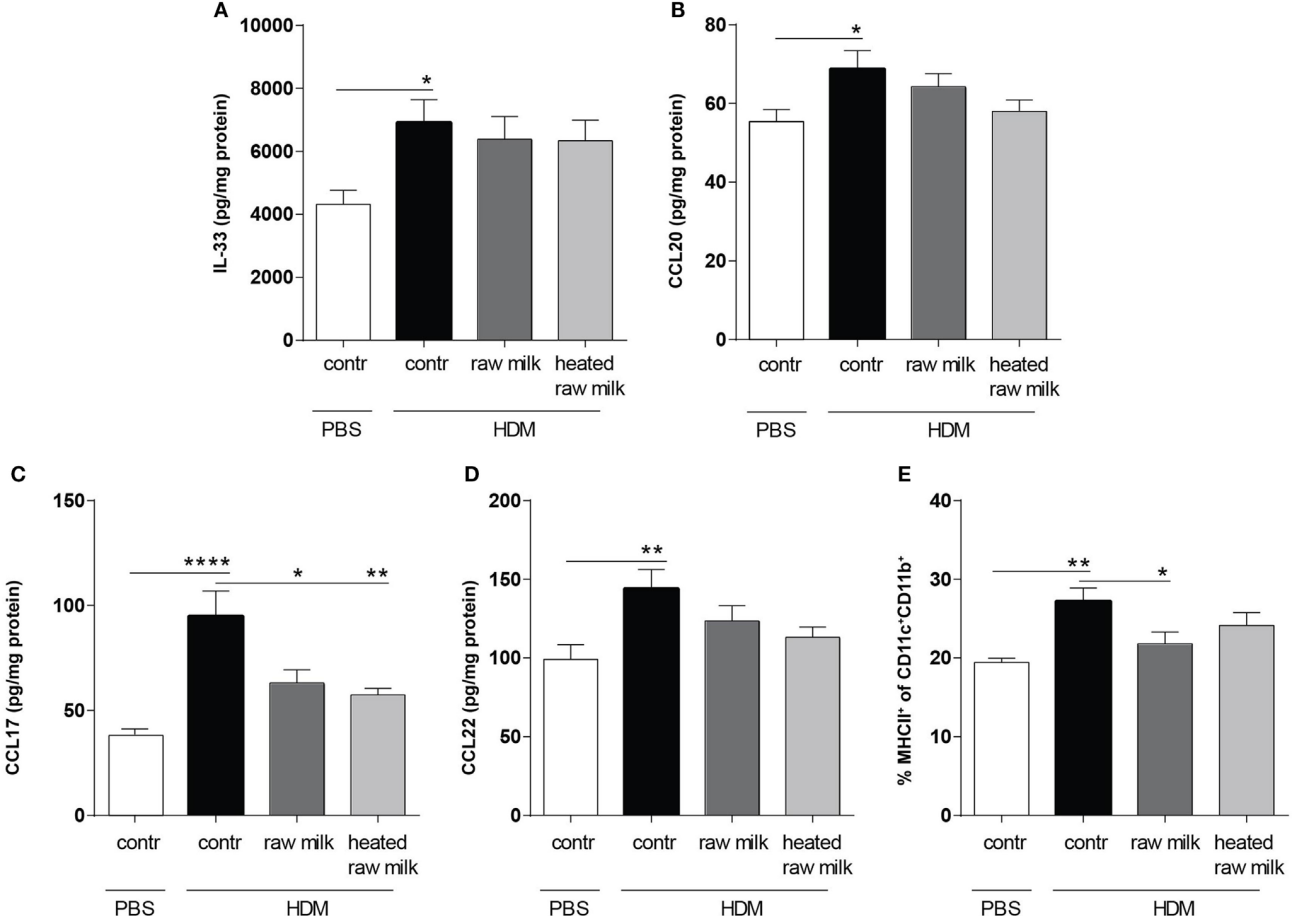
Frequency of CD11b^+^ conventional dendritic cells (cDCs) and related CCL17 concentrations in the lung were decreased after exposure to raw milk. Levels of epithelial- and DC-derived Th2-polarizing mediators in the lung. **(A)** IL-33, **(B)** CCL20, **(C)** CCL17, and **(D)** CCL22 concentrations were measured in supernatant of lung homogenates (picogram per milligram protein). **(E)** The percentage of CD11b^+^ cDCs (MHCII^+^ of CD11c^+^CD11b^+^ cells) was analyzed in lung cell suspensions. Data are presented as mean ± SEM, *n* = 8–9 mice/group. *P < 0.05, **P < 0.01, ****P < 0.0001 as analyzed with one-way ANOVA followed by Bonferroni’s multiple comparisons test. Contr, mice treated with PBS.

To determine the effect of raw and heated raw milk on pulmonary cytokines and chemokines, we analyzed supernatants of lung homogenates. IL-33, CCL20, CCL17, and CCL22 concentrations were increased in HDM-mice compared to PBS-mice (Figures 3A–D). Neither raw nor heated raw milk were able to affect the concentrations of IL-33, CCL20, and CCL22 (Figures 3A,B,D), but both significantly reduced the concentration of CCL17 (Figure 3C). Since CCL17 is mainly secreted by CD11b^+^ cDCs, this DC subtype was analyzed in lung cell suspensions. A higher percentage of CD11b^+^ cDCs was observed in HDM-mice compared to PBS-mice (Figure 3E). While heated raw milk showed no effect on this increase, it was reduced by raw milk (Figure 3E).

### Percentage of Th2 and Th17 Cells in the Lung Decreases after Exposure to Raw Milk

**Figure 4:**
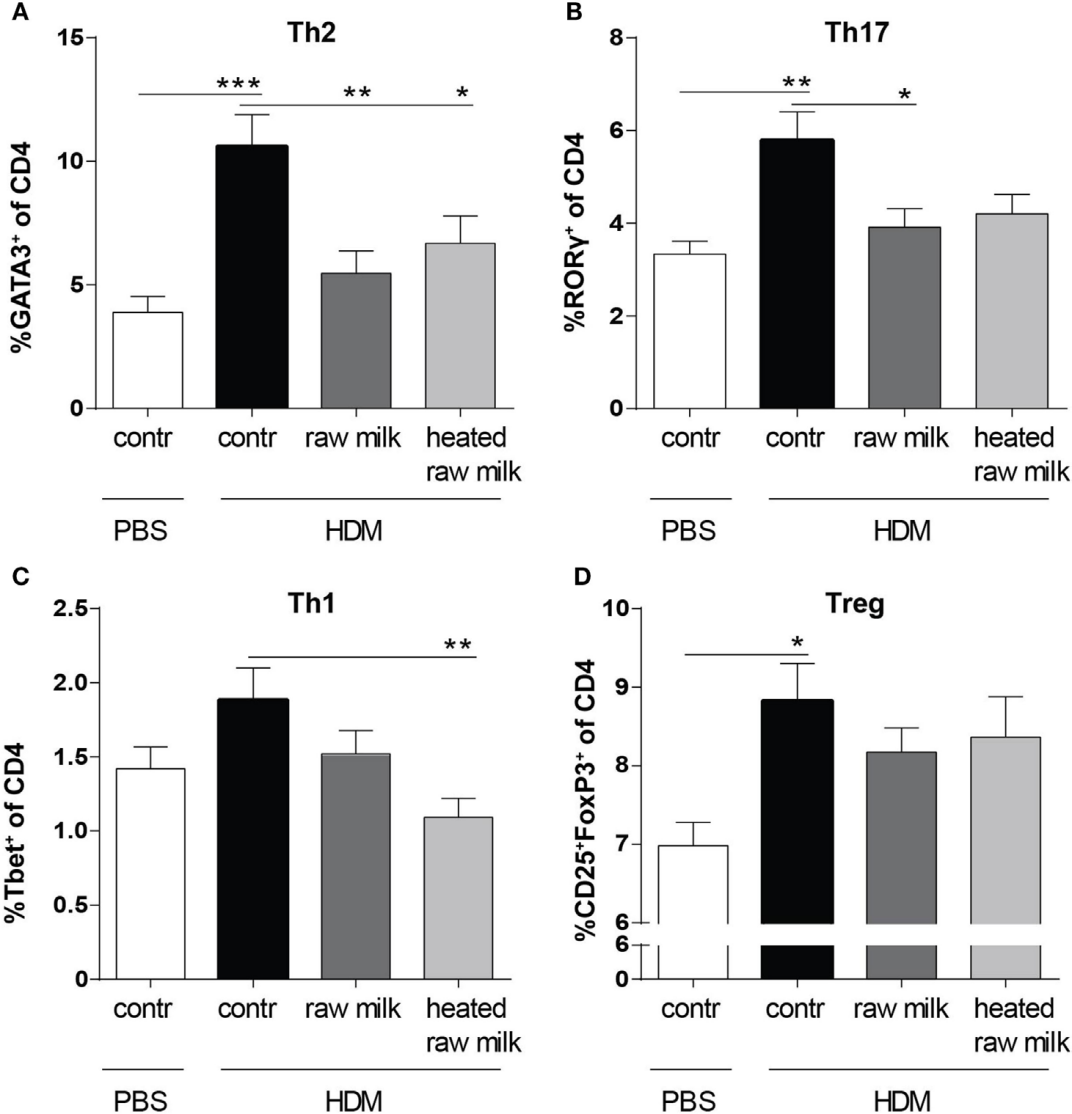
Raw milk suppressed the frequency of Th2 and Th17 cells in the lung. Lung T cell subsets. **(A)** The percentage of Th2 cells (GATA3^+^ of CD4^+^ cells), **(B)** Th17 cells (RORγ^+^ of CD4^+^ cells), **(C)** Th1 cells (Tbet^+^ of CD4^+^ cells), and **(D)** regulatory T cells (Tregs) (CD25^+^FoxP3^+^ of CD4^+^ cells) was analyzed in lung cell suspensions. Data are presented as mean ± SEM, *n* = 8–9 mice/group. *P < 0.05, **P < 0.01, and ***P < 0.001 as analyzed with one-way ANOVA followed by Bonferroni’s multiple comparisons test. Contr, mice treated with PBS.

Lung cell suspensions were analyzed for T cell subsets. The frequency of Th2, Th17, and regulatory T cells (Tregs) was higher in HDM-mice than in PBS-mice (Figures 4A,B,D). In line with the reduced concentration of the Th2 attracting chemokine CCL17, the frequency of Th2 cells in the lung was reduced by both raw and heated raw milk (Figure 4A). For Th17 cells, a similar effect was observed (Figure 4B), while neither milk type affected the percentage of Treg cells (Figure 4D). Th1 cells were not increased in HDM-mice compared to PBS-mice and showed no raw milk effect (Figure 4C). However, heated raw milk, did reduce the percentage of Th1 cells (Figure 4C).

### Raw Milk Reduces IL-4 and IL-13 Production after *Ex Vivo* Restimulation of Lung T Cells with HDM

**Figure 5:**
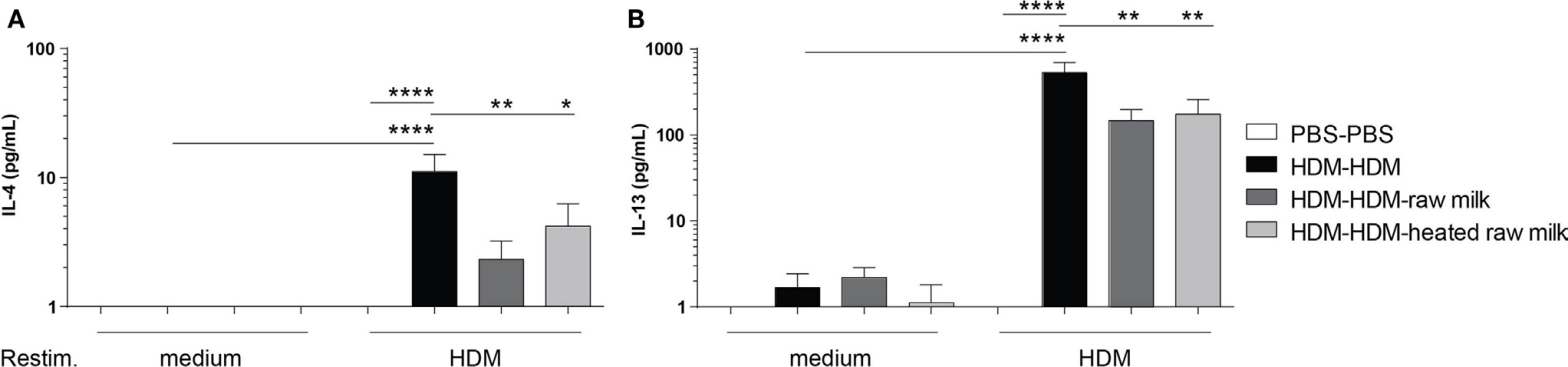
IL-4 and IL-13 production after *ex vivo* restimulation with house dust mite (HDM) was inhibited by raw milk. Lung cell suspensions were *ex vivo* restimulated with medium or HDM for 4 days (37°C, 5% CO_2_). **(A)** IL-4 (treatment: *P* < 0.01, restimulation: *P* < 0.001, interaction: *P* < 0.01) and **(B)** IL-13 (treatment: *P* < 0.01, restimulation: *P* < 0.001, interaction: *P* < 0.01) concentrations were measured in the supernatants (picogram per milliliter). Data are presented as mean ± SEM, *n* = 7–9 mice/group. *P < 0.05, **P < 0.01, ****P < 0.0001 as analyzed with two-way ANOVA followed by Bonferroni’s multiple comparisons test.

To investigate whether the reduction in Th2 and Th17 cell frequency by raw and heated raw milk also affected the production of Th2 and Th17 related cytokines, we measured allergen-specific IL-4, IL-13, and IL-17A secretion in supernatants of lung cell suspensions after *ex vivo* restimulation with HDM. No cytokine production was observed in PBS-mice, whereas HDM-mice showed a significant, HDM-specific, increase in IL-4 and IL-13 (Figures 5A,B). Raw milk and heated raw milk reduced the IL-4 and IL-13 concentrations (Figures 5A,B). Although IL-17A concentrations were increased in HDM-mice compared to PBS-mice, neither raw nor heated raw milk showed any effect (data not shown).

### Inhibition of Pulmonary IL-5 and IL-13 Concentrations by Raw Milk

**Figure 6:**
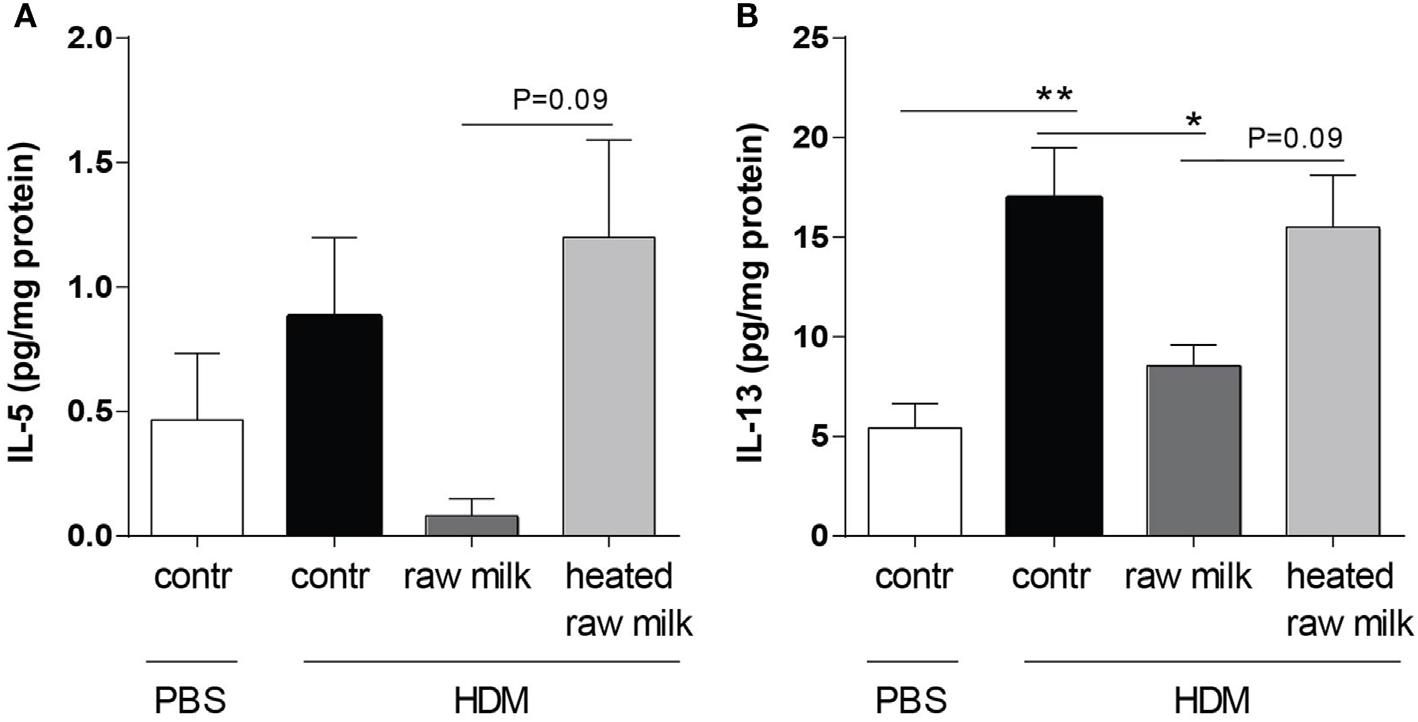
Raw milk reduced pulmonary IL-5 and IL-13 concentrations. Type 2 cytokine concentrations in the lung. **(A)** IL-5 and **(B)** IL-13 concentrations were measured in the supernatant of lung homogenates (picogram per milligram protein). Data are presented as mean ± SEM, *n* = 7–9 mice/group. *P < 0.05, **P < 0.01 as analyzed with Kruskal–Wallis test followed by Dunn’s multiple comparisons test (IL-5) or one-way ANOVA followed by Bonferroni’s multiple comparisons test (IL-13). Contr, mice treated with PBS.

Supernatants of lung homogenates were furthermore analyzed to investigate whether raw or heated raw milk also reduced local concentrations of type 2 cytokines. IL-5 concentrations did not differ between HDM-mice and PBS-mice, but IL-13 concentrations were increased (Figures 6A,B). This increase in IL-13 was reduced by raw milk (Figure 6B), which also seemed to suppress IL-5 concentrations (Figure 6A). No reduction in local IL-5 or IL-13 concentrations was observed when mice received heated raw milk (Figures 6A,B).

## Discussion

In the present study, we demonstrated that raw cow’s milk consumption prevented the development of asthma in a murine HDM-induced allergic asthma model. Strong protective effects on AHR and airway inflammation were observed which coincided with a reduced type 2 immune response. Heat-treated raw milk showed no asthma-protective effect.

Large-scale epidemiological studies in European populations have consistently shown that growing up on a traditional farm protects against the development of asthma (5–8). These studies invested a lot of effort to determine farm-related exposures contributing to this protective effect. Raw cow’s milk consumption is one of the factors repeatedly shown to be protective (9–13). However, all current evidence showing a relationship between raw cow’s milk consumption and the prevention of asthma is based on observed associations that do not confirm a causal relationship. To investigate causality, we used a murine HDM-induced allergic asthma model. This model mimics hallmark features of allergic asthma in humans and has clinical relevance due to the use of HDM as aeroallergen (20, 21).

As our data show, and in accordance with existing epidemiological studies, raw cow’s milk prevents the development of allergic asthma. Raw milk prevented HDM-induced AHR and abrogated pulmonary inflammation. This protective effect was abolished after heating the milk, which suggests that heat-sensitive milk components play a role in the asthma-protective effect of raw milk. The observed contribution of heat-sensitive components is in line with observations from the GABRIELA study, a large epidemiological study in which an inverse association between the whey fraction of the milk and asthma was found (9). Whey proteins such as α-lactalbumin, β-lactoglobulin, lactoferrin, immunoglobulins, and TGF-β are heat-sensitive and are known to have immunomodulatory effects (22, 23). Follow-up studies with these milk components need to demonstrate whether they also play a role in our model. Another constituent, previously found to be associated with a reduced asthma risk, is the fat content of raw milk (24). Since this fat content is unaffected by heat, it could be responsible for the slight reduction we observed in AHR by mice consuming heated raw milk (0.78 mg/mL of methacholine). However, for the moment, we can only speculate that the fat content in combination with heat-sensitive components is responsible for the asthma-protective effect of raw milk in our study.

To determine how raw milk exerts its effect, we looked at several inflammatory mediators involved in the pathogenesis of allergic asthma. Allergic asthma is generally induced by HDM allergens, and a key trigger in the recognition of HDM is the activation of epithelial cells (25). Epithelial cells are activated when HDM allergens bind to pattern recognition receptors (PRRs), such as TLR4, on their cell membrane. On activation, they produce chemokines and cytokines that attract DCs to the lung and instruct them to induce Th2 immunity (26). Since DCs also express PRRs themselves, they can also be directly activated by HDM allergens (1). Epithelial- (IL-33 and CCL20) and DC-derived (CCL17 and CCL22) mediators were indeed increased after HDM exposure in our model, and the frequency of CD11b^+^ cDCs was also elevated. These results are in line with human studies showing that IL-33, CCL20, CCL17, and CCL22 levels, as well as DC numbers, are elevated in the airways of asthmatic patients compared to healthy controls (27–30). Raw milk did not affect the epithelial mediators but did reduce the percentage of CD11b^+^ cDCs and the concentration of CCL17. The CD11b^+^ cDC subpopulation plays an important role in inducing allergic inflammation via the secretion of CCL17 and CCL22 (31). Both of these chemokines are CCR4 ligands and will attract CCR4-expressing Th2 cells to the lungs, which is essential for the development of allergic asthma (32). The importance of CCL17 and CCL22 is furthermore confirmed in murine asthma models where their neutralization with specific antibodies resulted in attenuation of AHR and airway inflammation (33, 34).

The reduction in the CD11b^+^ cDC-derived CCL17 concentration by raw milk may have resulted in a reduced Th2 cell influx. Indeed, the frequency of GATA3^+^CD4^+^Th2 cells in the lung was decreased by raw milk. GATA3 expression is known to be markedly increased in T cells derived from the airways of asthmatic patients compared to healthy individuals (35). It is essential not only for the differentiation of naïve T cells into Th2 cells but also for the secretion of Th2-related cytokines (IL-4, IL-5, and IL-13) (36, 37). In addition to reducing Th2 cell frequency, raw milk also reduced the percentage of Th17 cells in the lung, which mainly recruit and activate neutrophils and are known to be increased in asthmatic patients (38). This reduction in Th17 cells indeed coincided with a reduced neutrophil influx to the lung. While the exact contribution of Th17 cells in allergic asthma remains to be elucidated, blocking Th17 immunity as a therapy is gaining interest (39, 40). The reduction of Th2 and Th17 cells by raw milk was not accompanied by a shift toward a more Th1 or Treg immune response.

**Figure 7:**
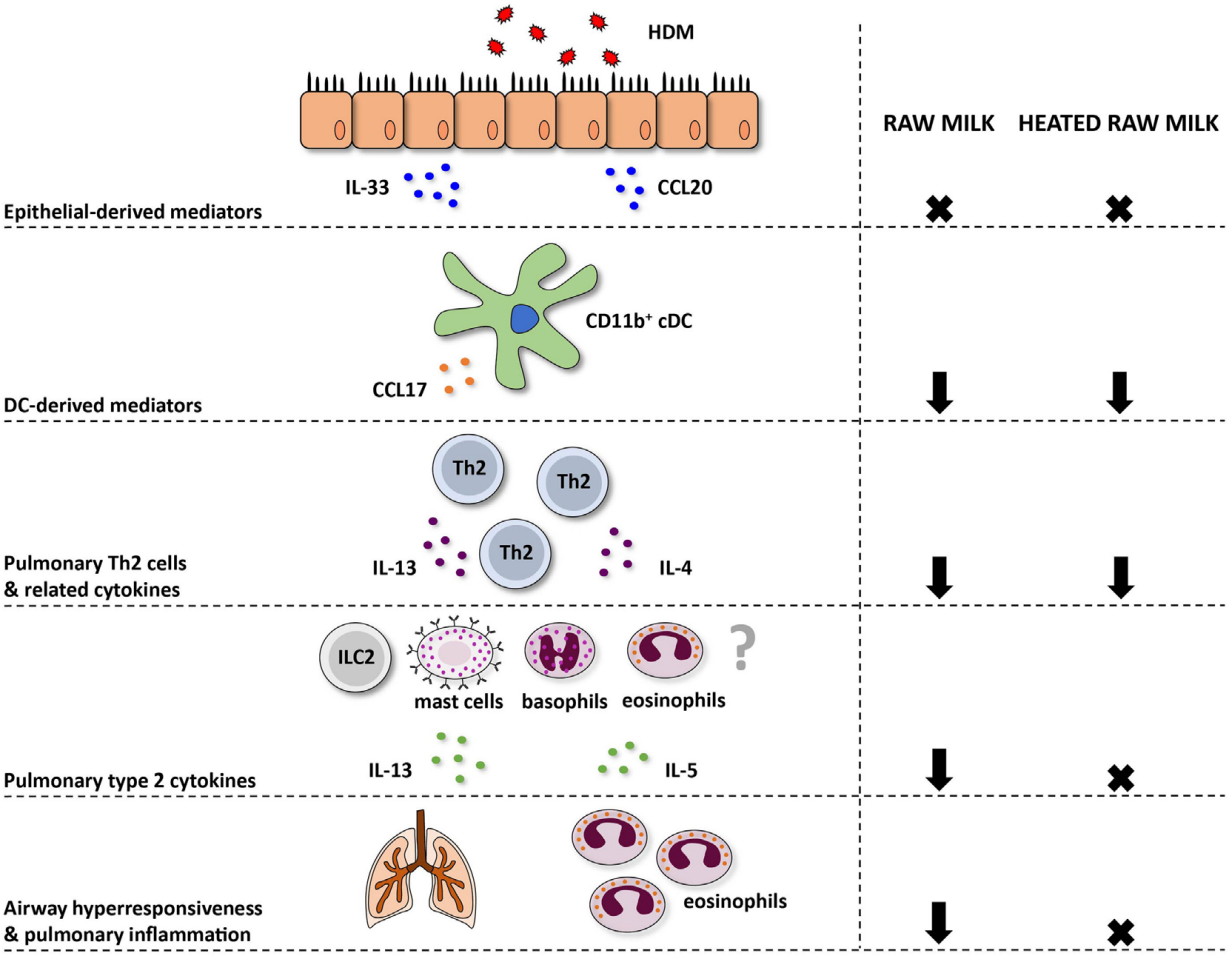
Schematic overview of the effects of raw and heated raw milk on allergic asthma. Following exposure to house dust mite (HDM), lung epithelial cells produce chemokines, like CCL20, that attract immature preconventional dendritic cells (cDCs) to the lung, and cytokines, like IL-33, that activate CD11b^+^ cDCs. Concentrations of these epithelial-derived mediators in the lung were unaffected by raw and heated raw milk. Activated lung CD11b^+^ cDCs subsequently migrate to the mediastinal lymph nodes where they differentiate naïve T cells into Th2 cells. These Th2 cells migrate back to the lung mucosal tissue in response to CCL17 and CCL22 produced by poorly migratory DCs. Lung CCL17 concentration, as well as lung Th2 cell frequency, was reduced by both milk types. On subsequent HDM challenges, Th2 cells will start to produce IL-4, IL-5, and IL-13. The concentration of these Th2-related cytokines was also suppressed by both milk types after *ex vivo* restimulation of lung T cells with HDM. However, pulmonary concentrations of these cytokines, measured in lung homogenates, were only reduced by raw milk. Since not only Th2 cells but also ILC2 cells, mast cells, basophils, and eosinophils can produce these type 2 cytokines, it suggests that only raw milk affects these other cells types. Eventually, only raw milk prevented airway hyperresponsiveness and pulmonary inflammation indicating the importance of reduced local type 2 cytokine levels.

In accordance with the reduced Th2 cell influx, raw milk also reduced concentrations of Th2 related cytokines. IL-4 and IL-13 production was suppressed after *ex vivo* restimulation of lung T cells with HDM. In addition, local pulmonary IL-13 concentrations were inhibited and a similar pattern was observed for IL-5 (Figure 7). Together, these type 2 cytokines are responsible for the salient features of allergic asthma (3, 41).

In contrast to raw milk, heated raw milk was not able to prevent the development of asthma. Even though it reduced pulmonary CCL17 concentrations, the frequency of Th2 and Th17 cells in the lung, and the IL-4 and IL-13 production by lung T cells after *ex vivo* restimulation with HDM, which is potentially beneficial in allergic diseases, this did not result in reduced airway inflammation in our study. Heated raw milk did not affect local pulmonary IL-5 and IL-13 concentrations. This discrepancy in the effects on type 2 cytokines might be explained by the fact that local levels can also be produced by immune cells other than T cells. It is well known that type 2 cytokines are produced not only by Th2 cells but also by ILC2 cells, eosinophils, basophils, and mast cells (42). Since raw milk suppressed local type 2 cytokine levels, this implies that a reduction in locally produced IL-5 and IL-13 is essential to the preventive effects of raw milk. Furthermore, it suggests that only raw milk affects these other cells types, but this needs to be confirmed in future studies (Figure 7).

The promising results of this study suggest that there is a causal relationship between raw cow’s milk consumption and the prevention of allergic asthma. The protective effect seems to start as early as the DC level, where mediators priming Th2 immunity were suppressed. However, suppression of local type 2 cytokine levels seems to be crucial. This study supports current epidemiological findings and emphasizes the need for minimally processed, safe milk. By comparing raw and heated raw milk, a first attempt was made to pinpoint the milk components underlying the asthma-protective effect of raw milk. Heat-sensitive milk components were found to be responsible. However, future research should aim at elucidating the specific heat-sensitive milk components involved and their underlying mechanism.

## Ethics Statement

This study was carried out in accordance with the recommendations of the Ethical Committee for Animal Research of the Utrecht University, Utrecht, The Netherlands. The protocol was approved by the Ethical Committee for Animal Research of the Utrecht University, Utrecht, The Netherlands (DEC2014.III.12.117).

## Author Contributions

SA, KV, and BE designed the study; SA, KV, and MD performed the animal experiment; SA, KV, MD, AL-M, and BE acquired the data; SA and KV analyzed and interpreted the data; SA and KV drafted the manuscript; BE, GH, TB, and JG critically revised the manuscript; all authors read and approved the final manuscript.

## Conflict of Interest Statement

This study is financially supported by Nutricia Research. GH, JG, and BE are (partly) employed at Nutricia Research. All other authors report no potential conflicts of interest.

## References

[1] Lambrecht BN Hammad H The immunology of asthma. Nat Immunol (2015) 16(1):45–56. 10.1038/ni.3049 25521684

[2] Holgate ST. Innate and adaptive immune responses in asthma. Nat Med (2012) 18(5):673–83. 10.1038/nm.2731 22561831

[3] Hammad H Lambrecht BN. Dendritic cells and epithelial cells: linking innate and adaptive immunity in asthma. Nat Rev Immunol (2008) 8(3):193–204. 10.1038/nri2275 18301423

[4] Alfvén T Braun-Fahrländer C Brunekreef B von Mutius E Riedler J Scheynius A Allergic diseases and atopic sensitization in children related to farming and anthroposophic lifestyle-the PARSIFAL study. Allergy (2006) 61(4):414–21. 10.1111/j.1398-9995.2005.00939.x 16512802

[5] Kilpeläinen M Terho EO Helenius H Koskenvuo M. Farm environment in childhood prevents the development of allergies. Clin Exp Allergy (2000) 30(2):201–8. 10.1046/j.1365-2222.2000.00800.x 10651772

[6] Riedler J Eder W Oberfeld G Schreuer M. Austrian children living on a farm have less hay fever, asthma and allergic sensitization. Clin Exp Allergy (2000) 30(2):194–200. 10.1046/j.1365-2222.2000.00799.x 10651771

[7] von Ehrenstein OS von Mutius E Illi S Baumann L Böhm O von Kries R Reduced risk of hay fever and asthma among children of farmers. Clin Exp Allergy (2000) 30(2):187–93. 10.1046/j.1365-2222.2000.00801.x 10651770

[8] Ernst P Cormier Y. Relative scarcity of asthma and atopy among rural adolescents raised on a farm. Am J Respir Crit Care Med (2000) 161(5):1563–6. 10.1164/ajrccm.161.5.9908119 10806155

[9] Loss G Apprich S Waser M Kneifel W Genuneit J Buchele G The protective effect of farm milk consumption on childhood asthma and atopy: the GABRIELA study. J Allergy Clin Immunol (2011) 128(4):766–73.e4. 10.1016/j.jaci.2011.07.048 21875744

[10] Waser M Michels KB Bieli C Flöistrup H Pershagen G von Mutius E Inverse association of farm milk consumption with asthma and allergy in rural and suburban populations across Europe. Clin Exp Allergy (2007) 37(5):661–70. 10.1111/j.1365-2222.2006.02640.x 17456213

[11] Ege MJ Frei R Bieli C Schram-Bijkerk D Waser M Benz MR Not all farming environments protect against the development of asthma and wheeze in children. J Allergy Clin Immunol (2007) 119(5):1140–7. 10.1016/j.jaci.2007.01.037 17349684

[12] Perkin MR Strachan DP. Which aspects of the farming lifestyle explain the inverse association with childhood allergy? J Allergy Clin Immunol (2006) 117(6):1374–81. 10.1016/j.jaci.2006.03.008 16751000

[13] Riedler J Braun-Fahrländer C Eder W Schreuer M Waser M Maisch S Exposure to farming in early life and development of asthma and allergy: a cross-sectional survey. Lancet (2001) 358(9288):1129–33. 10.1016/s0140-6736(01)06252-3 11597666

[14] von Mutius E Vercelli D Farm living: effects on childhood asthma and allergy. Nat Rev Immunol (2010) 10(12):861–8. 10.1038/nri2871 21060319

[15] Michalski MC. On the supposed influence of milk homogenization on the risk of CVD, diabetes and allergy. Br J Nutr (2007) 97(4):598–610. 10.1017/S0007114507657900 17349070

[16] Braun-Fahrländer C von Mutius E. Can farm milk consumption prevent allergic diseases? Clin Exp Allergy (2011) 41(1):29–35. 10.1111/j.1365-2222.2010.03665.x 21155907

[17] Kool M Willart MA van Nimwegen M Bergen I Pouliot P Virchow JC An unexpected role for uric acid as an inducer of T helper 2 cell immunity to inhaled antigens and inflammatory mediator of allergic asthma. Immunity (2011) 34(4):527–40. 10.1016/j.immuni.2011.03.015 21474346

[18] Verheijden KA Henricks PA Redegeld FA Garssen J Folkerts G. Measurement of airway function using invasive and non-invasive methods in mild and severe models for allergic airway inflammation in mice. Front Pharmacol (2014) 5:190. 10.3389/fphar.2014.00190 25161620 PMC4129625

[19] Verheijden KA Willemsen LE Braber S Leusink-Muis T Delsing DJ Garssen J Dietary galacto-oligosaccharides prevent airway eosinophilia and hyperresponsiveness in a murine house dust mite-induced asthma model. Respir Res (2015) 16:17. 10.1186/s12931-015-0171-0 25849971 PMC4327967

[20] Nials AT Uddin S. Mouse models of allergic asthma: acute and chronic allergen challenge. Dis Model Mech (2008) 1(4–5):213–20. 10.1242/dmm.000323 19093027 PMC2590830

[21] Gregory LG Lloyd CM. Orchestrating house dust mite-associated allergy in the lung. Trends Immunol (2011) 32(9):402–11. 10.1016/j.it.2011.06.006 21783420 PMC3381841

[22] Chatterton DE Nguyen DN Bering SB Sangild PT. Anti-inflammatory mechanisms of bioactive milk proteins in the intestine of newborns. Int J Biochem Cell Biol (2013) 45(8):1730–47. 10.1016/j.biocel.2013.04.028 23660296

[23] van Neerven RJ Knol EF Heck JM Savelkoul HF. Which factors in raw cow’s milk contribute to protection against allergies? J Allergy Clin Immunol (2012) 130(4):853–8. 10.1016/j.jaci.2012.06.050 22939757

[24] Brick T Schober Y Böcking C Pekkanen J Genuneit J Loss G Omega-3 fatty acids contribute to the asthma-protective effect of unprocessed cow’s milk. J Allergy Clin Immunol (2016) 137(6):1699–706.e13. 10.1016/j.jaci.2015.10.042 26792208

[25] Lambrecht BN Hammad H The airway epithelium in asthma. Nat Med (2012) 18(5):684–92. 10.1038/nm.2737 22561832

[26] Hammad H Chieppa M Perros F Willart MA Germain RN Lambrecht BN.House dust mite allergen induces asthma via toll-like receptor 4 triggering of airway structural cells. Nat Med (2009) 15(4):410–6. 10.1038/nm.1946 19330007 PMC2789255

[27] Saglani S Lui S Ullmann N Campbell GA Sherburn RT Mathie SA IL-33 promotes airway remodeling in pediatric patients with severe steroid-resistant asthma. J Allergy Clin Immunol (2013) 132(3):676–85. 10.1016/j.jaci.2013.04.012 23759184 PMC4218948

[28] Pichavant M Charbonnier AS Taront S Brichet A Wallaert B Pestel J Asthmatic bronchial epithelium activated by the proteolytic allergen Der p 1 increases selective dendritic cell recruitment. J Allergy Clin Immunol (2005) 115(4):771–8. 10.1016/j.jaci.2004.11.043 15805997

[29] Pilette C Francis JN Till SJ Durham SR. CCR4 ligands are up-regulated in the airways of atopic asthmatics after segmental allergen challenge. Eur Respir J (2004) 23(6):876–84. 10.1183/09031936.04.00102504 15219001

[30] Möller GM Overbeek SE Van Helden-Meeuwsen CG Van Haarst JMW Prens EP Mulder PG Increased numbers of dendritic cells in the bronchial mucosa of atopic asthmatic patients: downregulation by inhaled corticosteroids. Clin Exp Allergy (1996) 26(5):517–24. 10.1111/j.1365-2222.1996.tb00571.x 8735863

[31] Gill MA. The role of dendritic cells in asthma. J Allergy Clin Immunol (2012) 129(4):889–901. 10.1016/j.jaci.2012.02.028 22464668

[32] Barnes PJ The cytokine network in asthma and chronic obstructive pulmonary disease. J Clin Invest (2008) 118(11):3546–56. 10.1172/JCI36130 18982161 PMC2575722

[33] Kawasaki S Takizawa H Yoneyama H Nakayama T Fujisawa R Izumizaki M Intervention of thymus and activation-regulated chemokine attenuates the development of allergic airway inflammation and hyperresponsiveness in mice. J Immunol (2001) 166(3):2055–62. 10.4049/jimmunol.166.3.2055 11160256

[34] Gonzalo J-A Pan Y Lloyd CM Jia G-Q Yu G Dussault B Mouse monocyte-derived chemokine is involved in airway hyperreactivity and lung inflammation. J Immunol (1999) 163(1):403–11. 10384142

[35] Caramori G Lim S Ito K Tomita K Oates T Jazrawi E Expression of GATA family of transcription factors in T-cells, monocytes and bronchial biopsies. Eur Respir J (2001) 18(3):466–73. 10.1183/09031936.01.00040701 11589343

[36] Rayees S Malik F Bukhari SI Singh G. Linking GATA-3 and interleukin-13: implications in asthma. Inflamm Res (2014) 63(4):255–65. 10.1007/s00011-013-0700-6 24363163

[37] Barnes PJ Immunology of asthma and chronic obstructive pulmonary disease. Nat Rev Immunol (2008) 8(3):183–92. 10.1038/nri2254 18274560

[38] Zhao Y Yang J Gao YD Guo W. Th17 immunity in patients with allergic asthma. Int Arch Allergy Immunol (2010) 151(4):297–307. 10.1159/000250438 19844129

[39] Cosmi L Liotta F Maggi E Romagnani S Annunziato F. Th17 cells: new players in asthma pathogenesis. Allergy (2011) 66(8):989–98. 10.1111/j.1398-9995.2011.02576.x 21375540

[40] Ivanov S Linden A. Interleukin-17 as a drug target in human disease. Trends Pharmacol Sci (2009) 30(2):95–103. 10.1016/j.tips.2008.11.004 19162337

[41] Kudo M Ishigatsubo Y Aoki I Pathology of asthma. Front Microbiol (2013) 4:263 10.3389/fmicb.2013.00263 24032029 PMC3768124

[42] Schuijs MJ Willart MA Hammad H Lambrecht BN. Cytokine targets in airway inflammation. Curr Opin Pharmacol (2013) 13(3):351–61. 10.1016/j.coph.2013.03.013 23643194

